# Mapping of heterologous expressed sequence tags as an alternative to microarrays for study of defense responses in plants

**DOI:** 10.1186/1471-2164-10-273

**Published:** 2009-06-18

**Authors:** Alexander M Boutanaev, Olga A Postnikova, Lev G Nemchinov

**Affiliations:** 1 Institute of Basic Biological Problems, Russian Academy of Sciences, 2 Institute Street, Pushchino, Moscow Region, 142292, Russia; 2 USDA/ARS, Plant Sciences Institute, Molecular Plant Pathology Laboratory, Beltsville MD 20705, USA

## Abstract

**Background:**

Microarray technology helped to accumulate an immense pool of data on gene expression changes in response to different environmental factors. Yet, computer- generated gene profiling using expressed sequence tags (EST) represents a valuable alternative to microarrays, which allows efficient discovery of homologous sequences in evolutionarily different species and comparison of gene sets on the whole genome scale. In this study, we used publicly available EST database derived from different plant species infected with a variety of pathogens, to generate an expression profile of homologous genes involved in defense response of a model organism, *Arabidopsis thaliana*.

**Results:**

EST-driven prediction identified 4,935 genes (16% of the total *Arabidopsis *genome) which, according to the origin of EST sets, were associated with defense responses in the reference genome. Profiles of defense-related genes, obtained by mapping of heterologous EST, represent putative *Arabidopsis *homologs of the corresponding species. Comparison of these profiles in pairs and locating common genes allowed estimating similarity between defense-related gene sets of different plant species. To experimentally support computer data, we arbitrarily selected a number of transcription factor genes (TF) detected by EST mapping. Their expression levels were examined by real-time polymerase chain reaction during infection with yellow strain of *Cucumber mosaic virus*, a compatible virus systemically infecting *Arabidopsis*. We observed that 65% of the designated TF were upregulated in accordance with the EST-generated profile.

**Conclusion:**

We demonstrated that heterologous EST mapping may be efficiently used to reveal genes involved in host defense responses to pathogens. Upregulated genes identified in this study substantially overlap with those previously obtained by microarrays.

## Background

Currently, a prevailing view is that land plants coevolved with pathogens since their first emergence on earth, 500–700 million years ago [1]. This coevolution created complex relationships involving various mechanisms of plant defenses and diverse strategies used by pathogens to overcome these barriers [2].

A comprehensive analysis and comparison of gene repertoires associated with host-pathogen interactions are of significant interest. A difficulty of this task, however, lies in the fact that nucleotide sequences and functional annotation of plant genomes are at different stages of completion. Therefore, a straight sequence comparison of homologous genes belonging to distinct genomes and displaying differential activity is challenging. Heterologous EST mapping, allowing indirect evaluation of similarity between gene sets of various genomes, represents an alternative solution [3]. Using this approach, we show that gene expression changes in response to diverse biological agents may be evolutionarily conserved in higher plant species and such conservation involves a wide range of inducible genes composing defense-related regulatory networks and up-regulation of genes controlling general plant metabolic pathways.

## Results

### Profiling of pathogenesis-related genes in Arabidopsis

For heterologous EST mapping we used the most representative EST sets of wheat (*Triticum aestivum*), tomato (*Lycopersicum esculentum*), potato (*Solanum tuberosum*), and soybean (*Glycine max*) infected with various plant pathogens (57,855 EST) as well as EST from the same species (approximately 42,000 EST) that were not infected with any microorganisms (see Additional file 1). Among pathogenic species were fungi, bacteria, and viruses (10, 4 and 2 respectively). Using the BLAST search engine [4] together with custom-developed EST-mapping software, that attributes each EST from the database to a gene in the annotated *Arabidopsis *genome [3], we mapped "infectious" and "healthy" EST of each origin (tomato, potato, wheat and soybean) to *A*. *thaliana *genome, which resulted in several expression profiles of *Arabidopsis *genes corresponding to the four selected genomes. "Healthy" expression profiles were sequentially deduced from the "infectious" profiles of the same genomes and therefore served as filters to eliminate genes that do not participate in defense response. Resultant individual profiles of defense-related *A. thaliana *genes corresponding to the four other plant species were added together to yield a generalized profile, reflecting a global pattern of *A. thaliana *gene expression in response to infection. Furthermore, at the next level of selection and using the same technique of profile building, this previously obtained comprehensive profile was additionally filtered through 114,249 EST derived from *A. thaliana *"healthy" libraries. Eventually, a final expression profile of 4935 genes (16% of the genome) presumably participating in defense response of *A. thaliana *and exhibiting positive profile values (elevated expression level) has been created.

We used individual *Arabidopsis *gene expression profiles obtained in the first round of selection to evaluate similarity between defense-related gene sets of corresponding species. This was done by locating common genes in pairs of the resultant expression profiles of *Arabidopsis*. Generally, a number of upregulated genes in a profile depends on a quantitative representation of EST libraries, therefore a number of common genes had to be normalized by the sum of "infection-related" EST derived from a pair of analyzed species. We obtained the following normalized values: *S. tuberosum*/*S. lycopersicum *(0.05), *S. tuberosum*/*G. max *(0.04), *S. lycopersicum*/*G. max *(0.04), *T. aestivum*/*S. tuberosum *(0.01), *T. aestivum*/*S. lycopersicum *(0.01), *T. aestivum*/*G. max *(0.01). These numbers have been mostly identical among dicotyledonous species and at least four times lower between the monocotyledon wheat and any of the four dicots.

Since *Arabidopsis *has the most comprehensive functional annotation of any plant genome, we were able to join pathogenesis-related genes identified by expression profiling into functional groups (see Additional file 2). These groups could further be characterized by a degree of their engagement in defense reactions as well as by the average expression level of constituent genes.

**Figure 1:**
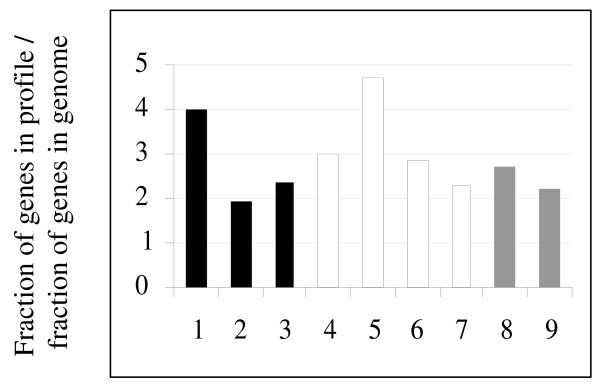
**Involvement of different functional groups of *Arabidopsis *genes in host response against pathogens**. Genes related to the general plant metabolism (black shaded columns): 1 – photosynthesis, 2 – mitochondrion, 3 – protein synthesis. Abiotic stress-responsive genes (clear columns): 4 – oxidative stress, 5 – cold, 6 – heat, 7 – water deprivation. Biotic stress-responsive genes (grey columns): 8 – fungi, 9 – bacteria. The ordinates represent a ratio between a fraction of functionally related genes in profile and a fraction of functionally related genes in the whole genome i.e. overrepresentation of the genes in the functional group as compared to the whole genome.

The contribution of each individual functional group to defense reaction was evaluated as follows. First, we found what fraction of genes is attributed to the group on the scale of the profile. Next, we found what fraction of genes is attributed to the group on the scale of the whole genome. Finally, a ratio between the first value and the second one showed possible involvement of each of the groups in defense response (Figure 1).

In general, the ratio indicates overrepresentation in the expression profile if greater than 1.0, or underrepresentation, if less than 1.0. In other words, this is a degree to which different functional groups of *Arabidopsis *genes related to stress responses and to general plant metabolism are involved in defense reactions. Although both abiotic and biotic stress response genes contributed considerably to the expression profile, degree of such contribution varied from 2.3 (water deprivation) to 4.7 (cold) in case of abiotic stress, whereas in case of biotic stress it was 2.2 (bacteria) and 2.7 (fungi), respectively. Interestingly, genes of general plant metabolism were also substantially involved in defense against pathogen challenge. Implication of photosynthesis-related genes was especially high (4.0) whereas involvement of mitochondrial genes and genes related to protein synthesis was 1.9 and 2.4 respectively.

We next compared average values of expression levels in groups of functionally related genes with that of the whole profile (Table 1). The average values for the genes related to photosynthesis, oxidative stress and cold were significantly higher than the average value for the whole profile while the corresponding values for the rest of the functional groups were not.

**Table 1:** Mean values of expression level in groups of functionally related genes as compared to the mean profile value.

Functional group	Mean profile value	*p*-value
Photosynthesis	2.24 ± 1.06	0.0006
Mitochondrion	0.36 ± 0.07	0.160
Translation	0.60 ± 0.55	0.166
**Response to**:		
oxidative stress	0.56 ± 0.18	0.013
cold	1.03 ± 0.56	0.008
heat	0.28 ± 0.07	0.110
water deprivation	0.55 ± 0.31	0.094
fungi	0.41 ± 0.11	0.099
bacteria	0.48 ± 0.18	0.059

**Total profile**	0.32 ± 0.02	

Among other notable groups of genes related to defense response were protein kinases and transcription factors (TF). We found that among 294 kinases, 74 (25%) were related to endomembrane system with 23 annotated to encode membrane receptors and 4 described as defense response genes (see Additional file 2). Among 293 TF 34 (12%) were annotated as genes responsive to both abiotic and biotic stimuli.

### Experimental assessment of computer-generated expression profile

**Figure 2:**
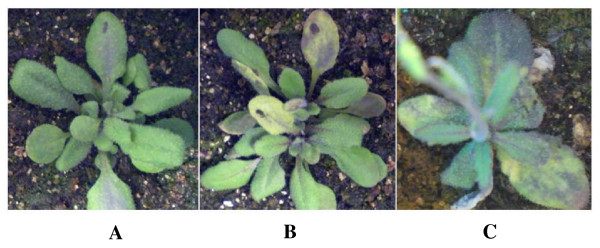
**Symptoms on CMV(Y)-infected *A. thaliana *plants ecotype Col-0**. A, Mock-inoculated plant. B, CMV(Y)-infected plant, 3 dpi. C, CMV(Y)-infected plant, 7 dpi.

To support EST-driven predictions, we took advantage of a previously described experimental model, which is based on the interaction between *A. thaliana *and its compatible pathogen, yellow strain of *Cucumber mosaic virus *(CMV (Y)) [5]. CMV(Y)-infected *A. thaliana *plants ecotype Columbia 0 (Col-0) developed chlorotic symptoms on inoculated leaves 3 days post inoculation (dpi). Symptoms became systemic 5 to 7 dpi (Figure 2). Virus presence in the inoculated *Arabidopsis *plants was confirmed by reverse transcription polymerase chain reaction (RT-PCR) with CMV-specific primers (not shown).

**Figure 3:**
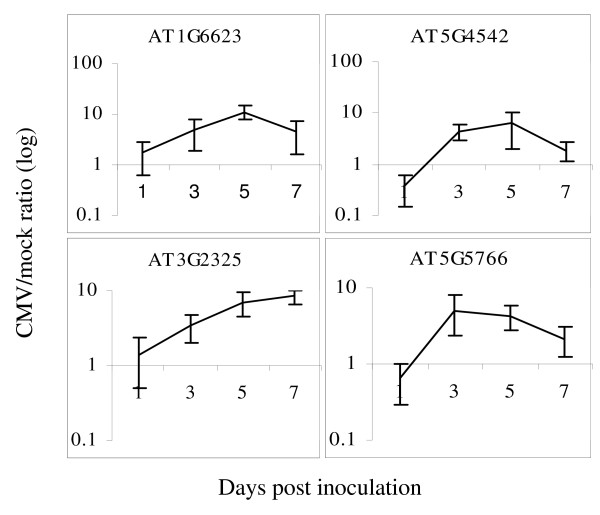
**Activation of *Arabidopsis *TF genes during viral infection**. The ratio between infected and control plants (CMV(Y)/mock) represents gene expression level. Maximum expression level of four TF genes was 5 to 11 times greater in infected plants as compared to healthy plants. Bars represent confidence intervals (p = 0.05).

Expression of the genes encoding TF was of special interest because of their basic role in regulating plant responses to environmental stresses. Since the entire expression profile varied from 0.001 to 41.906 with an average of 0.326, we have arbitrarily selected 20 TF with profile values in the range of 0.1–2.0, which approximately represented the middle of the profile. Out of twenty studied TF, thirteen (65%) had elevated expression levels in response to the viral infection (see Additional file 3). Figure 3 shows some of the TF, upregulated in response to CMV(Y). A complete overlap between computer data and its experimental validation could hardly be expected since expression profiles were deduced from plants infected with a variety of different pathogens whereas our model system included a single plant virus. Needless to say, these data demonstrate a high stringency of computer-generated gene profiling even when applied to a random model of host-pathogen interaction.

### Comparison between computer-generated profile and microarray analysis of gene expression changes

To quantitatively compare the depth of the pathogen response repertoire found by a computer-generated suit of upregulated genes with *Arabidopsis *pathogen array data, we used the Shannon index, which is frequently employed to measure diversity in categorical data. Global gene expression changes assessed by the Affymetrix ATH1 array in *A. thaliana *plants infected with *Plum pox virus *(PPV) [6] were used as a representative example of pathogen array experiment. The whole genome ATH1 chip contains 22810 genes, which allowed us to compare these data with our global profile.

The index values were 7.7 in the case of EST mapping and 7.4 in the microarray experiment. However, for the genes encoding transcription factors, index values calculated from EST mapping were higher when compared to microarray data (5.6 and 4.7, respectively). This indicates a similarity of gene coverage between whole sets of induced genes identified by EST mapping and microarrays. It also points out the advantage of EST-generated profiling for uncovering low-expression level genes, such as TF.

In both cases (microarray and EST mapping), the total amount of common induced genes was 1028, which corresponded to 51% and 21%, respectively (2013 upregulated genes in microarray and 4935 upregulated genes in EST mapping). In the microarray experiment, a number of defense-related genes specifically responsive to PPV pathogenesis in *Arabidopsis *plants were found to be induced. Since corresponding EST, obtained from PPV-infected *Arabidopsis*, were not used to build a computer-generated profile, it is not surprising that the same genes were not revealed by EST mapping. On the other hand, the low-expression level genes were likely underrepresented in the microarray.

## Discussion

Microarray technology helped to accumulate an immense pool of data on gene expression changes in response to different environmental factors. Yet, a computer-generated gene profiling, although limited by availability of EST collections in databases, represents a valuable alternative to microarrays, which allows efficient discovery of homologous sequences in evolutionarily different species and comparison of gene sets on the whole genome scale. Importantly, computer profiling is especially sensitive to low-abundance transcripts that are normally underrepresented in cellular mRNA, such as transcription factors. Furthermore, the method does not require a significant statistical support because of a large number of available transcripts (tens of thousand EST) and reliable selection of upregulated genes in expression profile. Previously, we successfully used computer-assisted gene profiling for identification of testis-specific genes in *Drosophila *[3]. Thus, an expansion of data obtained by microarrays with the help of EST-based gene profiling as well as comparative analysis of information collected by these two approaches would undoubtedly be beneficial. Indeed, our quantitative comparison of gene profiling data using the Shannon index demonstrated that EST mapping and microarrays can efficiently complement each other.

Application of computer-assisted gene profiling toward study of gene expression induced by plant pathogens was the main goal of this study. In general, our results are in a good agreement with those generated by genome chip technology. As a whole, two independent approaches (microarray and EST profiling) reveal the same classes of functionally related genes with expression levels elevated as a result of defense response. These are many groups of genes involved in general plant metabolism (photosynthesis, protein synthesis, mitochondrial genes) along with the genes of host stress and defense responses [6-8]. In the EST-derived profile, genes annotated as defense-related and responsive to abiotic stress represented 20% and 23%, respectively, of all the stress-responsive genes in the *Arabidopsis *genome. Other classes found included putative membrane-associated receptors and many transcription factors with unknown regulatory functions.

According to our estimate, contribution of different groups of functionally-related genes to plant defense responses varied significantly. A share of genes associated with photosynthesis, which under the same conditions is a rather constitutive and stable process, was especially high (Table 1). This may indicate their role in basal defense responses rather than a rapid switch toward viral needs [9]. Other researchers also found considerable variation in the degree of involvement of different groups of genes in host responses [6].

In addition, computer-generated profile of gene expression changes during pathogen attack uncovered activation of many genes normally induced in response to abiotic factors. Engagement and expression levels of cold stress-responsive genes were the largest. Even though it is hard to suggest their specific role in defense reactions, it is known that plant defense mechanisms may be temperature-dependent. For instance, at low temperatures RNA silencing-mediated antiviral defense is inhibited [10].

The experimental system of compatible virus-host interaction between *A. thaliana *and CMV(Y), which was used in this work to evaluate reliability of computer-generated profiling, is based on the fact that the plant is not equipped with powerful means of genetic resistance such as the presence of *R *genes and therefore will not develop hypersensitive response or systemic acquired resistance to the pathogen. Observed expression changes in the genes, pre-selected according to the created profiles, demonstrated high stringency of EST profiling even when applied to a random model of host-pathogen interaction. Activation of transcription factors, genes of general metabolism and stress genes may correspond to the initial, basal response to the pathogen, which is eventually overtaken by infection.

Profiles of defense-related genes, obtained by mapping of heterologous EST, represent putative *Arabidopsis *homologs of the corresponding species. Comparison of these profiles in pairs and locating common genes allowed indirect estimation of the similarity between defense-related gene sets of different plant species based on *Arabidopsis *homologs. This similarity was high among three dicotyledonous species and rather low in monocotyledonous *T. aestivum *vs three dicots. This suggests that the repertoire of genes participating in defense reactions in dicots and monocots, although similar to some degree, has nevertheless considerably diverged. It is, apparently, generally conserved in dicots. Since *Arabidopsis *(*Brassicales*) and *Glycine max *(*Fabales*) are in the Rosids and both *Lycopersicum esculentum *and *Solanum tuberosum *(*Solanaceae) *are in the Asterids, it appears that evolutionary conservation of defense responses in these two groups may be traced to their common ancestor as far as 150 million years ago [11]. Differences in defense mechanisms between dicots and monocots could in theory date back to their split ~200 million years ago [11,12].

In summary, we have demonstrated that computer-assisted gene profiling based on heterologous EST mapping may be efficiently used to reveal genes involved in host defense responses to pathogens. Unlike microarrays, it permitted indirect comparison of the complete sets of functionally related genes in different species on the whole genome scale. The method allows effective identification of tissue-specific and organ-specific genes, genes expressed at a particular developmental stage, and genes responsive to internal and external stimuli. Moreover, EST profiling permits to further narrow down these groups to individual biological processes, cellular compartments, or molecular functions. For instance, in this work, EST mining identified a large group of defense-related genes encoding calcium-binding proteins. Since calcium is an important second messenger, playing an essential role in plant defence responses [13,14], these data may eventually be applied to identify a role of Ca^2+^-binding proteins in specific plant-pathogen interactions.

For the quantitative representation of expression level in profile, only EST derived from non-normalized cDNA libraries can be used, even though low-level expression genes in such libraries are underrepresented. On the contrary, in normalized libraries where specific genes are better represented, their EST cannot be used for quantitative analysis of expression level.

Presently, EST mining can easily be adopted to reveal specific genes in *Arabidopsis*, *Drosophila*, and mouse genomes and, to a lesser degree, in the human genome. Databases such as Genbank are constantly supplied with new EST and annotated genome sequences. Soon, vast datasets will be available from deep RNA sequencing on next generation platforms. Because of this, we believe that the approach conveyed in this study may be successfully applied to further increase our knowledge and understanding of transcriptom dynamics in general and more specifically, its role in host-pathogen interactions and plant defense mechanisms.

## Conclusion

We demonstrated that heterologous EST mapping may be efficiently used to reveal genes involved in host defense responses to pathogens. Upregulated genes identified in this study substantially overlap with those previously obtained by microarrays. As a whole, two independent approaches (microarray and computer generated gene profiling) reveal the same classes of functionally related genes with expression levels elevated as a result of defense response. These include groups of genes involved in general plant metabolism (photosynthesis, protein synthesis, mitochondrial genes) alongside genes of host stress and defense responses.

## Methods

### EST mapping and building of gene expression profiles

Gene profiling was performed using a software package that attributes each EST from the database to a gene in the annotated *Arabidopsis *genome and generates a table with the numbers of EST found for each gene [3]. Briefly, it consisted of the three following steps: 1. BLAST homology search in the annotated genome using EST derived from different sources (organs, tissues, stages, physiological states of an organism). 2. Assignment of each EST to a corresponding gene based on coordinates of the homology region present in the BLAST output file. The step also includes building of an expression profile by assigning each gene a fraction of homologous EST that is, a value of expression level. 3. Finally, in order to obtain specific expression profile, a number of profiles originated from different EST sets are subtracted from the profile in question.

### Databases

The following publicly available databases were used: Genbank at National Center for Biotechnology Information  and The Arabidopsis Information Resource .

### Computing the Shannon index

Shannon diversity index representing information entropy is used to measure diversity in categorical data. It is computed from this formula:



*ni *– The profile value of gene *i*.

*N *– The sum of all profile values.

*pi *= *ni*/*N *– The relative "abundance" of each gene.

*S *– The number of genes.

### Plant growth, virus purification and inoculation of plants

*Arabidopsis thaliana *plants (ecotype Columbia-0, accession Col-0/Redei-L206440) were obtained from Lehle Seeds, Round Rock, TX, USA and grown in 6-in. square pots at a density of 2–3 plants per pot to 21 days of age, in a Percival growth chamber (model E30BHOC8, Percival Scientific, Perry, IA, USA), set for a 16-h photoperiod and 24°C. A yellow strain of CMV was obtained from Dr. H. Takahashi, Department of Life Science, Graduate School of Agricultural Science, Tohoku University, Sendai, Japan. The virus was propagated in tobacco *Nicotiana benthamiana *and purified by differential centrifugation as described by Lot et al. [15] with minor modifications. The quality of the purified viral preparation was additionally confirmed by transmission electron microscopy and Western blotting with CMV(Y) specific antibodies (a gift of H. Takahashi) (not shown). Virions were resuspended to approximately 0.5 mg/ml in 20 mM potassium phosphate buffer (pH 7.2) and 10 μl of the solution were rub-inoculated onto leaves of three weeks-old plants marked and dusted with carborundum. Control plants were mock inoculated with phosphate buffer alone. Five non-inoculated (systemic) leaves on each of 3 plants per time point were harvested at 1, 3, 5, and 7 dpi, snap frozen in liquid nitrogen, and stored at 80°C until RNA extraction.

### RNA extraction, first-strand synthesis and real-time RT-PCR

Total RNA was extracted using TRIzol reagent as described by manufacturer (Invitrogen Corp., Carlsbad, CA, USA). Copy DNA was synthesized using SuperScript First-Strand cDNA Synthesis System according to manufacturer's directions (Invitrogen). Real-time PCR was performed with iQ SYBR Green Supermix kit (Bio-Rad Laboratories, Inc., Hercules, CA, USA) on the MiniOpticon Real-Time PCR system (Bio-Rad) using the following parameters: 94°C – 1 min (1 cycle); 94°C – 30 sec, 60°C – 30 sec, 72°C – 30 sec (30 cycles). Amplification was performed in two biological and two technical replicas. *Arabidopsis *actin gene *ACTIN1 *(*AT2G37620*) was used as a reference in all real-time PCR experiments.

### Primers design

Primers for real-time PCR were designed using sequences of the last two exons of each gene (when possible) to insure amplification of the mRNA only. On average, length of the amplified fragments was 200–300 nts. (see Additional file 4).

## Abbreviations

EST: expressed sequenced tags; TF: transcription factors; CMV (Y): *Cucumber mosaic virus*, yellow strain; *ACTIN 1*: structural constituent of cytoskeleton

## Authors' contributions

AMB and LGN designed research; AMB, OAP and LGN performed research; AMB and LGN analyzed research; AMB and LGN wrote the manuscript.

## Supplementary Material

Additional file 1 **Species, pathogens and EST origins.** Click here for file

Additional file 2 **Functional groups of pathogenesis-related genes identified by expression profiling.** Click here for file

Additional file 3 ***Arabidopsis *transcription factors activated in response to CMV (Y) infection.** Click here for file

Additional file 4 **Primers used for real time PCR.** Click here for file
